# A systematic review of vision and vision-language foundation models in ophthalmology

**DOI:** 10.1016/j.aopr.2025.10.004

**Published:** 2025-10-24

**Authors:** Kai Jin, Tao Yu, Gui-shuang Ying, Zongyuan Ge, Kelvin Zhenghao Li, Yukun Zhou, Danli Shi, Meng Wang, Polat Goktas, Andrzej Grzybowski

**Affiliations:** a Eye Center of the Second Affiliated Hospital, Zhejiang University School of Medicine, Hangzhou, China; b Zhejiang Provincial Key Laboratory of Ophthalmology, Zhejiang Provincial Clinical Research Center for Eye Diseases, Zhejiang Provincial Engineering Institute on Eye Diseases, Hangzhou, China; c Zhejiang University Chu Kochen Honors College, Hangzhou, China; d Center for Preventive Ophthalmology and Biostatistics, Department of Ophthalmology, Perelman School of Medicine, University of Pennsylvania, Philadelphia, PA, USA; e AIM for Health Lab, Faculty of IT, Monash University, Australia; f Department of Data Science and AI, Faculty of IT, Monash University, Australia; g Department of Ophthalmology, Tan Tock Seng Hospital, Singapore; h Center for AI in Medicine, Lee Kong Chian School of Medicine, Nanyang Technological University, Singapore; i Byers Eye Institute, Stanford, Palo Alto, CA, USA; j Institute of Ophthalmology, University College London, London, UK; k Hawkes Institute, University College London, London, UK; l NIHR Biomedical Research Centre at Moorfields Eye Hospital NHS Foundation Trust, London, UK; m School of Optometry, The Hong Kong Polytechnic University, Kowloon, Hong Kong, China; n Research Centre for SHARP Vision (RCSV), The Hong Kong Polytechnic University, Kowloon, Hong Kong, China; o Centre for Innovation and Precision Eye Health, Yong Loo Lin School of Medicine, National University of Singapore, Singapore; p Department of Ophthalmology, Yong Loo Lin School of Medicine, National University of Singapore, Singapore; q Faculty of Engineering and Natural Sciences, Sabanci University, Türkiye; r Institute for Research in Ophthalmology, Foundation for Ophthalmology Development, Poznan, Poland; s Department of Ophthalmology, University of Warmia and Mazury, Olsztyn, Poland

**Keywords:** Ophthalmology, Vision foundation models, Vision-language models, Artificial intelligence, Clinical integration

## Abstract

**Background:**

Vision and vision-language foundation models, a subset of advanced artificial intelligence (AI) frameworks, have shown transformative potential in various medical fields. In ophthalmology, these models, particularly large language models and vision-based models, have demonstrated great potential to improve diagnostic accuracy, enhance treatment planning, and streamline clinical workflows. However, their deployment in ophthalmology has faced several challenges, particularly regarding generalizability and integration into clinical practice. This systematic review aims to summarize the current evidence on the use of vision and vision-language foundation models in ophthalmology, identifying key applications, outcomes, and challenges.

**Main text:**

A comprehensive search on PubMed, Web of Science, Scopus, and Google Scholar was conducted to identify studies published between January 2020 and July 2025. Studies were included if they developed or applied foundation models, such as vision-based models and large language models, to clinically relevant ophthalmic applications. A total of 10 studies met the inclusion criteria, covering areas such as retinal diseases, glaucoma, and ocular surface tumor. The primary outcome measures are model performance metrics, integration into clinical workflows, and the clinical utility of the models. Additionally, the review explored the limitations of foundation models, such as the reliance on large datasets, computational resources, and interpretability challenges.

The majority of studies demonstrated that foundation models could achieve high diagnostic accuracy, with several reports indicating excellent performance comparable to or exceeding those of experienced clinicians. Foundation models achieved high accuracy rates up to 95% for diagnosing retinal diseases, and similar performances for detecting glaucoma progression. Despite promising results, concerns about algorithmic bias, overfitting, and the need for diverse training data were common. High computational demands, EHR compatibility, and the need for clinician validation also posed challenges. Additionally, model interpretability issues hindered clinician trust and adoption.

**Conclusions:**

Vision and vision-language foundation models in ophthalmology show significant potential for advancing diagnostic accuracy and treatment strategies, particularly in retinal diseases, glaucoma, and ocular oncology. However, challenges such as data quality, transparency, and ethical considerations must be addressed. Future research should focus on refining model performance, improving interpretability and generalizability, and exploring strategies for integrating these models into routine clinical practice to maximize their impact in clinical ophthalmology.

## 1 Introduction

Recent advancements in artificial intelligence (AI), particularly through the development of vision and vision-language foundation models, have demonstrated great potential to transform medical applications.^1^ These models, which encompass large language models (LLMs) and vision-based architectures, have shown substantial promise across various healthcare domains, with ophthalmology emerging as one of the key beneficiaries of these applications.^2^^,^^3^ Ophthalmology, with its high dependence on image-based diagnostics, complex decision-making processes, and increasing demand for automation, therefore offers an ideal landscape for the integration of AI technologies.^4^^,^^5^

Vision and vision-language foundation models are pretrained on large heterogeneous datasets comprising millions of images and paired image–text corpora, spanning both medical and non-medical sources. The pretraining process often leverages self-supervised learning paradigms such as masked auto encoding, contrastive learning, or cross-modal alignment (e.g., CLIP-like architectures), which enable the extraction of both fine-grained visual representations (e.g., retinal microstructures, vascular patterns) and higher-order semantic features relevant to clinical interpretation. These models can subsequently be fine-tuned for ophthalmic applications using transfer learning or parameter-efficient strategies like adapter tuning and prompt engineering, thereby reducing the demand for large, domain-specific annotated datasets. A defining characteristic is their cross-task transferability: the same backbone can seamlessly support tasks ranging from fundus and Optical Coherence Tomography (OCT) segmentation to disease classification, prognosis modeling, and even ophthalmic report generation. These models possess cross-task transferability and multimodal capabilities, enabling them to handle both image and language tasks. Unlike task-specific models, they can adapt to various ophthalmic applications, often achieving performance comparable to traditional machine learning (ML) methods while improving efficiency and adaptability. This adaptability is largely driven by their ability to leverage shared representations across domains, where features learned from general visual patterns can be repurposed for disease-specific recognition in ophthalmic imaging.^6^^,^^7^ In ophthalmology, these models are increasingly explored for diagnosing and predicting the prognosis of retinal diseases, glaucoma, and other ocular conditions.^8^^,^^9^ They are also employed to predict patient outcomes, automate routine tasks, and support clinical decision-making, thereby enhancing the efficiency of ophthalmic care.^10^

Despite the promising results, the widespread adoption of foundation models in clinical practice is hindered by several challenges.^11^ These include concerns about the interpretability of models, biases in training data, and the generalizability of models across diverse patient populations. Furthermore, integrating these models into existing healthcare infrastructures and ensuring reliable real-time performance in clinical settings remain a significant hurdles.^12^

This systematic review aims to systematically explore the potential applications of vision and vision-language foundation models in ophthalmology by synthesizing current literature. It examines the effectiveness of these models in improving diagnostic accuracy, optimizing treatment plans, and enhancing clinical workflows. Additionally, this review addresses limitations and challenges faced by these AI-driven tools and provides recommendations for future research and clinical implementation.

By evaluating the current state of vision and vision-language foundation models in ophthalmology, this review seeks to provide a comprehensive overview of their role in modernizing ophthalmic care, highlighting both their potential and the need for cautious, evidence-based integration into clinical practice.

## 2 Methods

This systematic review was conducted to evaluate the application, performance, and challenges associated with vision and vision-language foundation models in ophthalmology. The review followed the Preferred Reporting Items for Systematic Reviews and Meta-Analyses (PRISMA) guidelines to ensure transparency and reproducibility.

### 2.1 Eligibility criteria

Studies were included in the review if they met the following criteria: (1) The study applied a foundation model, in clinical ophthalmology; (2) The model was used for diagnosis, prediction for incidence or progression, or treatment planning of eye diseases (e.g., retinal diseases, glaucoma); (3) The study was published between January 2020 and July 2025 in peer-reviewed journals, with full-text paper available in English. This period was chosen as this period marked a significant increase in the application and development of foundation models in the medical field.

Exclusion criteria were: (1) Studies that did not use foundation models or did not focus on ophthalmology; (2) Study papers that were reviews, opinions, or case reports; (3) Studies that did not report on model performance or clinical outcomes.

### 2.2 Information sources and search strategy

A comprehensive literature search was conducted on July 7th, 2025, in several electronic databases, including PubMed, Web of Science, Scopus, and Google Scholar. Google Scholar was used only for forward/backward citation chasing rather than as a primary database. Boolean operators (AND, OR) were used to refine the search strategy. Specifically, "Foundation model" AND ("deep learning" OR "artificial intelligence") were combined to focus on studies integrating advanced AI methods. Terms related to the medical domain, such as "ophthalmology" AND ("retinal diseases" OR "ocular diseases"), were used to ensure the inclusion of relevant clinical research. To ensure the comprehensiveness of the search, "glaucoma" was included as a separate term, combined with "ocular diseases" using the OR operator. The search was restricted to articles published between January 1st, 2020 (the period marking the emergence of large-scale pretraining and multi-task transfer in medicine), and July 7th, 2025, to ensure the relevance and timeliness of the literature.

### 2.3 Study selection

After removing duplicates, titles and abstracts were screened by two independent reviewers (KJ and TY) to identify studies that met the eligibility criteria (Table S1). Full-text articles of potentially relevant studies were assessed for inclusion. Any disagreements between reviewers were resolved by consensus or by consulting a third reviewer (AG).

### 2.4 Data extraction

Data was extracted from the included studies using a standardized data extraction form. The following information was collected: Study characteristics (author(s), year of publication, study design), model type (e.g., deep learning, large language models (LLMs)), application area (e.g., retinal disease diagnosis, glaucoma monitoring), data used for training and validation (e.g., clinical images, datasets), performance metrics (e.g., diagnostic accuracy, sensitivity, specificity, area under the curve (AUC)), limitations and challenges discussed in the study (e.g., data quality, model interpretability).

### 2.5 Quality Assessment

The quality of the included studies was assessed using the QUADAS-2 (Quality Assessment of Diagnostic Accuracy Studies) tool, which evaluates the risk of bias in diagnostic accuracy studies.^13^ Each study was independently assessed by two reviewers (KJ and TY), with disagreements resolved by consensus. We added a summary table (Table S2) and a heatmap of domain-level ratings (Fig. 3).

### 2.6 Data synthesis

The data were qualitatively and quantitatively synthesized, and a narrative summary was provided. Due to the heterogeneous nature of the included studies, a meta-analysis was not performed. Key themes and findings regarding the applications of foundation models in ophthalmology, performance outcomes, and limitations were identified and discussed.

### 2.7 Outcome measures

The outcome measures of this review included performance metrics of the models, their integration into clinical workflows and clinical utility; limitations and challenges related to the integration of foundation models into clinical practice, such as data quality, interpretability, and bias.

## 3 Results

**Fig. 1:**
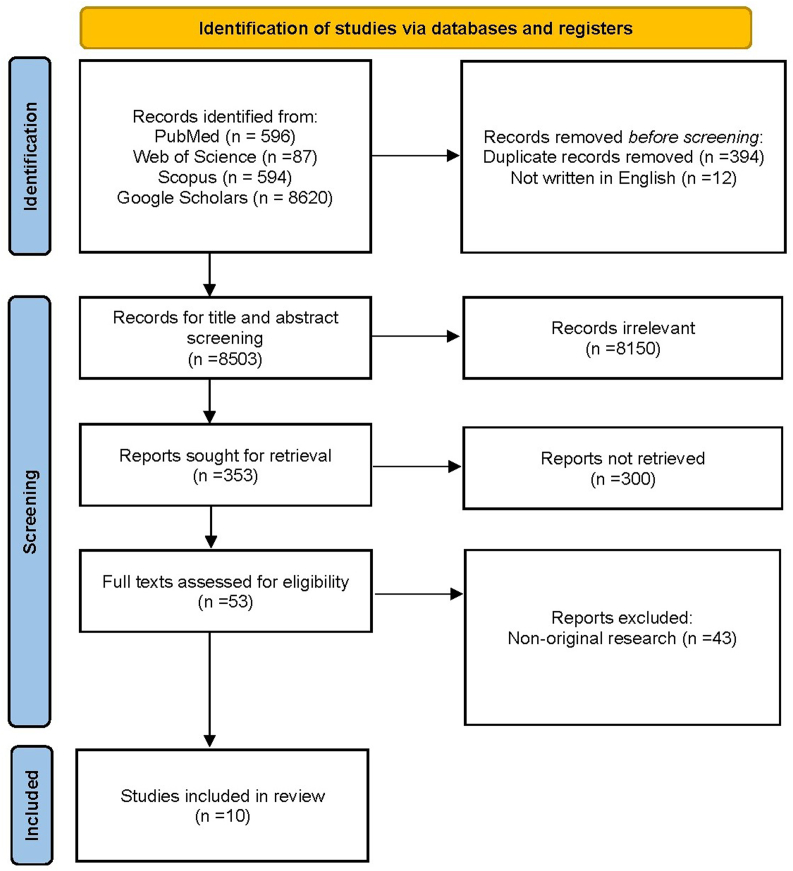
PRISMA 2020 flow diagram for this systematic review.

A total of 10 studies met the inclusion criteria and were included in this systematic review (Fig. 1). These studies collectively demonstrate the growing application of vision-based and vision-language foundation models, including both large language and vision-based models in various ophthalmological domains. The focus areas included retinal diseases, glaucoma, and ocular surface tumors (OSTs), with significant methodological diversity and performance outcomes.

### 3.1 Study characteristics

All included studies reviewed were published between 2023 and 2025, reflecting the rapid advancement of foundation model development in recent years (Table 1). All of these studies trained the foundation models using large datasets. Six studies utilized publicly available datasets (e.g., the EyePACS dataset, which contains 288,307 images), while four studies used institution-specific proprietary dataset, often integrating imaging with clinical data such as electronic health records (EHRs).^14^^,^^15^ The model training scale was substantial: for instance, VisionFM was trained on 3.4 million multimodal images, and MetaGP incorporated 8 million EHRs, indicating a trend toward large-scale pretraining for generalizability (Table 1).

**Table 1:** Characteristics of 10 foundation models for application in ophthalmology. AUC (Area Under the Curve), DR (Diabetic Retinopathy), Wet-AMD (Wet Age-Related Macular Degeneration), OCT (Optical Coherence Tomography), EHR (Electronic Health Records), CXR (Chest X-Ray), CT (Computed Tomography), MRI (Magnetic Resonance Imaging), F1 Score (F1 Score), IIR (Image-Image Retrieval), ITR (Image-Text Retrieval), OST (Ocular Surface Tumor).

Author	Publication Year	Model Name	Sample Size	Objective	Data Types	Main Findings	Clinical Significance	Study Design	External Validation	Geography
Yukun Zhou et al.^15^	2023	RETFound	1.6 million unlabelled Fundus images	For generalizable disease detection from Fundus images	OCT, fundus images	RETFound improves diagnostic accuracy with fewer labeled data	Facilitates faster adaptation to diverse medical tasks	Cross-sectional	Yes (EyePACS)	Global
Julio Silva- Rodríguez et al.^20^	2024	FLAIR	38 open-access datasets, 288,307 images	Enhance retinal fundus image analysis using domain-specific knowledge	Fundus images with textual descriptions	FLAIR outperforms task-specific models with expert knowledge integration	Enhances disease recognition accuracy in fundus images	Retrospective cohort	Yes (AREDS)	Multi-center (International)
Qiu et al.^19^	2024	VisionFM	3.4 million images from 500,000+ individuals	Develop a multimodal multitask AI model for ophthalmic use	Ophthalmic images across various modalities	VisionFM shows generalization and diagnostic accuracy across multiple modalities	Transforms clinical AI by enabling multimodal task adaptability	Cohort study	Yes (DR cohort, AMD cohort)	Multi-center (Global)
Danli Shi et al.^42^	2025	EyeCLIP	2.77 million images from 11 modalities	Develop a multimodal model for computational ophthalmology	Multimodal ophthalmic images, clinical text	EyeCLIP shows robust performance in disease classification and few-shot learning	Improves early detection of eye diseases with multimodal AI	Cross-sectional	Yes (MESSIDOR, UK Biobank)	Multi-center (Global)
Yuanyuan Peng et al.^21^	2025	FMUE	102,468 OCT images	Improve AI reliability in OCT-based retinal disease diagnosis	OCT images	FMUE enhances clinical AI reliability, achieving excellent performance on OCT data	Increases diagnostic reliability in clinical environments	Cross-sectional	Yes (External OCT)	Local
Fei Liu et al.^14^	2025	MetaGP	8 million EHRs, biomedical literature	Develop a model integrating medical records and imaging for diagnostics	EHRs, fundus images, chest X-rays (CXR) and CT scans	MetaGP boosts diagnosis of rare and emergent diseases	Improves clinician decision-making, especially in rare disease diagnosis	Cohort study	Yes (Composite rare-disease benchmark)	Multi-center (Global)
Jinzhuo Wang et al.^16^	2024	MINIM	200k ​+ ​paired images across 6 modalities	Build a self-improving generative model to synthesize high-fidelity medical images from text	OCT, fundus images, chest X-ray, chest CT, brain MRI, breast MRI	MINIM generates realistic images and improves diagnosis and mutation prediction	Enables non-invasive, AI-guided detection of HER2/EGFR mutations	Prospective cohort	Yes (Internal)	Multi-center (Global)
Yuqi Sun et al.^17^	2025	RETFound-DE	1 million synthetic images, 16.7% real data	Develop a data-efficient strategy for building medical foundation models	Fundus images, chest X-ray images	RETFound-DE shows competitive performance with synthetic data	Increases model performance with limited data	Cross-sectional	Yes (DR cohort, Glaucoma)	Global
Meng Wang et al.^18^	2025	RetiZero	341896 fundus images	Enhance diagnostic accuracy in fundus diseases with AI models	Fundus images	RetiZero enhances diagnostic accuracy in rare fundus diseases	Improves diagnostic accuracy in clinical settings	Cohort study	Yes (H1/H2/H3 external datasets)	Multi-center (Global)
Zhongwen Li et al.^22^	2025	OSPM	0.76 million ocular surface images	Create a model for detecting malignant and premalignant ocular tumors	Ocular surface images	OSPM improves accuracy in detecting malignant and premalignant OSTs	Enhances early detection and treatment of ocular tumors	Cross-sectional	Yes (JEH external dataset)	Multi-center (Global)

### 3.2 Application and model performance

*Retinal Diseases*: The majority of studies (n ​= ​8) focused on the diagnosis of retinal diseases, including diabetic retinopathy (DR) (n ​= ​8), age-related macular degeneration (AMD) (n ​= ​8), and diabetic macular edema (DME) (n ​= ​3). Vision-based models demonstrated high diagnostic performance.^16^, ^17^, ^18^, ^19^, ^20^, ^21^

• RETFound achieved AUC ​= ​0.94 for DR on the EyePACS dataset (N ​= ​288307, internal validation, prevalence ​≈ ​22%) and AUC ​= ​0.86 for wet AMD on the AREDS dataset (N ​= ​12532, external validation, prevalence ​≈ ​19%).
• RetiZero reported top-5 accuracy of 75.6% in detecting over 400 rare fundus conditions (N ​= ​342000, multi-ethnic, external validation).
• FLAIR and RETFound-DE showed strong performance even under few-shot and zero-shot scenarios (DR detection, sensitivity 0.91, specificity 0.89), reflecting their capacity for efficient adaptation with limited labeled data.

*Glaucoma Detection*: Three studies explored the use of foundation models for glaucoma detection, with models such as EyeCLIP and RETFound-DE reporting AUCs values between 0.721 and 0.913 (external dataset).^15^ RETFound-DE achieved AUC ​= ​0.902 for glaucoma on REFUGE-2 (external validation), with sensitivity 0.89 and specificity 0.86 ​at the pre-specified operating threshold. RETFound-DE demonstrated robust performance in both few-shot and cross-domain generalization tasks, supporting deployment across diverse clinical environments.

*Ocular Surface Tumors*: Two studies examined the application of AI models for detecting malignant and premalignant OSTs. The OSPM model achieved outstanding AUCs scores between 0.986 and 0.993, validating its potential as a screening tool in oncology ophthalmology (internal and external validation against histopathology gold standard).^22^ The model also maintained accuracy across external datasets such as JEH (Dataset or Clinical Site), highlighting its generalizability.

*Multimodal and Rare Disease Integration:* Several studies, such as MetaGP, extended foundation model capabilities to rare and urgent conditions through multimodal integration of fundus imaging, EHRs, and CT (Computed Tomography)/CXR (Chest X-Ray) scans. MetaGP yielded a diagnostic score of 1.57, outperforming GPT-4 (0.93) in rare disease classification, which reflects the model's performance in rare disease classification tasks by combining EHR data and imaging.^14^

For retinal diseases, the foundation models demonstrated sensitivity values ranging from 0.88 to 0.96 and specificity values from 0.85 to 0.93 (n ​= ​8), indicating strong diagnostic performance. In glaucoma detection, sensitivity ranged from 0.89 to 0.94, while specificity ranged from 0.84 to 0.91, refers to the three studies. Notably, several models maintained high performance when evaluated on external validation datasets, highlighting their robustness and generalizability across diverse clinical populations and settings. A detailed summary of model performance is presented in Table 2.

**Table 2:** Summary of foundation model performance in ophthalmology. AUC (Area Under the Curve), DR (Diabetic Retinopathy), Wet-AMD (Wet Age-Related Macular Degeneration), OCT (Optical Coherence Tomography), OCTDL (Optical Coherence Tomography Deep Learning), F1 Score (F1 Score), EHR (Electronic Health Records), IIR (Image-Image Retrieval), ITR (Image-Text Retrieval), JEH (Dataset or Clinical Site), MMAC (Multi-class Multi-label Classification), FIVES (Dataset for Retinal Disease Classification).

Model Name	Application Focus	Validation Dataset	Key Performance Metrics	Supervised Finetune	Few-shot	Zero-shot
RETFound	Disease detection from fundus images (CFP & OCT)	EyePACS (Internal)	AUC 0.94 (DR), AUC 0.86 (Wet-AMD), AUC 0.79 (Heart Failure)	AUC 0.943 (DR), 0.822 (IDRID)	Good performance with 50 ​% labeled data	AUC 0.754 (Ischemic Stroke), 0.669 (Parkinson's Disease)
		AREDS (External)				
FLAIR	Retinal image understanding via vision-language pre-training	20 ​× ​3 (Internal)	Accuracy 98.3% (20 ​× ​3), 66.7% (ODIR200 ​× ​3), 40% (MMAC)	AUC 0.602 (DR), AUC 0.918 (Glaucoma)	Significant improvement in few-shot settings with linear probe	33% (20 ​× ​3), 20% (ODIR200 ​× ​3, MMAC) improvement
		ODIR200 ​× ​3 (External)				
		MMAC (External)				
VisionFM	Multimodal AI for ophthalmology (8 imaging modalities)	Internal aggregate (Internal)	AUC 0.950 (Internal), AUC 0.945 (DR), AUC 0.974 (AMD)	AUC 0.950 (Internal)	Dice 77.54% (Few-shot OCT segmentation)	AUC 0.945 (DR)
		DR cohort (External)				
		AMD cohort (External)				
EyeCLIP	Multimodal ophthalmic visual-language model	MESSIDOR (External)	AUC 0.681–0.757 (DR), AUC0.684–0.721 (Glaucoma), AUC 0.800 (OCTID)	AUC 0.835 (DR), 0.913 (Glaucoma), 0.993 (OCTDL)	Outperformed others in few-shot tasks	AUC 0.681–0.757 (DR),0.684–0.721 (Glaucoma)
		UK Biobank/REFUGE-2 (External)				
		OCTID (External)				
FMUE	OCT-based retinal disease diagnosis with uncertainty estimation	Internal OCT (Internal)	F1 95.7%, AUC 0.989 (internal)	F1 97.23% (Internal), 99.16% (External)	Good performance with 1–16 labeled examples	Top-1 36%, Top-5 75.6% (rare disease diagnosis)
		External OCT (External)				
MetaGP	EHR & retinal imaging integration for rare/urgent disease diagnosis	Composite rare-disease benchmark (External)	Diagnostic score 1.57 (rare diseases)	Diagnostic score 1.57 (rare diseases)	Outperformed GPT-4 with 10–20% labeled data	Mean accuracy 0.698, F1 0.754 (rare disease diagnosis)
MINIM	OCT retinal disease diagnosis with synthetic data augmentation	Internal (Internal)	F1 93.0%, AUC 0.973	Top-1 79.7% (OCT), Top-1 86.0% (Fundus)	Performed well in few-shot settings	IIR: 62.25% (OCT), 64.83% (Fundus), ITR: 43.41% (OCT), 49.93% (Fundus)
RETFound-DE	Disease diagnosis with synthetic-data pretraining	DR cohort (External)	AUC 0.958 (DR), 0.732 (Glaucoma)	AUC ∼ 0.82 (few-shot)	AUC ∼ 0.82 with 2–16 samples per class	AUC 0.84 (cross-domain generalization)
		Glaucoma (External, REFUGE-2)				
RetiZero	Vision-language model for 400+ retinal diseases	Three clinical datasets of retinal photographs H1/H2/H3 (External)	AUC 0.997 (H1), AUC 0.980 (H2), AUC 0.993 (H3)	AUC 0.967 (H1), AUC 0.859 (H2), AUC 0.942 (H3)	Top-5 accuracy 75.6% (52 diseases)	Top-1 accuracy 0.442, Top-5 accuracy 0.840 (EYE-15)
OSPM	Ocular surface tumor classification (malignant, premalignant, benign)	Multicenter internal (Internal)	AUC 0.986–0.993 (internal)	AUC 0.986 (Malignant), 0.993 (Benign)	Better performance with 35–50% labeled data	AUC 0.694–0.940 (JEH external dataset)

### 3.3 Clinical integration and real-world deployment

Six of the included studies discussed efforts toward the integration of foundation models into clinical workflows. While all models demonstrated promising diagnostic results, none have yet achieved full deployment in routine ophthalmic practice. However, some models have been tested in pilot studies or limited-scope deployments. For instance, MetaGP has been used in real-world settings to integrate EHR and imaging data for diagnosing rare diseases, showing promising results in clinical trials for urgent care situations. Similarly, VisionFM has been tested in specific clinical environments for glaucoma and diabetic retinopathy screening. These examples highlight the translational potential of foundation models, particularly when compared to traditional task-specific AI models, which are already deployed for routine clinical tasks such as diabetic retinopathy screening.

**Fig. 2:**
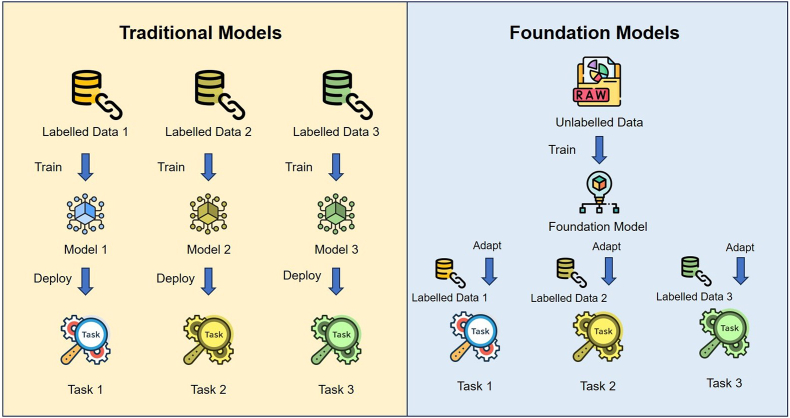
Diagram for traditional models and foundation models. This diagram highlights the advantages of foundation models over traditional models in generalizability, label efficiency, and computational efficiency. Unlike traditional models, a single foundation model can adapt to multiple tasks with less labeled data, making fine-tuning more efficient than training separate models from scratch.

Nonetheless, the findings suggest considerable translational potential, particularly when contrasted with traditional task-specific AI models. As illustrated in Fig. 2, foundation models offer distinct advantages over conventional architectures. Unlike traditional models that require separate training for each task using isolated labeled datasets, foundation models leverage large-scale pretraining on unlabelled data and can be adapted across multiple tasks with minimal labeled supervision. This results in improved generalizability, label efficiency, and computational scalability. Despite their promise, several technical and implementation-related challenges persist, as detailed below.

• *Computational Resource Demands*: Advanced models such as VisionFM, which incorporates multimodal imaging from over 500000 individuals across 3.4 million images, require substantial GPU-intensive infrastructure for both training and inference.^19^ While such models achieved AUCs up to 0.974 (AMD) and 0.945 (DR), real-time deployment in resource-constrained clinical environments remains a limitation without edge optimization or cloud-based support.
• *EHR Interoperability*: MetaGP, which integrates structured EHR data with multimodal images for rare disease diagnosis, demonstrated an average diagnostic score of 1.57, significantly outperforming GPT-4 (0.93).^14^ However, integration into diverse and often fragmented hospital information systems presents significant interoperability and data privacy challenges.
• *Clinician Validation and Interpretability Needs*: Even models with strong internal test results such as FMUE (focused on OCT-based retinal disease diagnosis with built-in uncertainty estimation) with F1 score of 95.7% and AUC of 0.989, necessitate clinicians involvement for results validation. Similarly, EyeCLIP, which achieved AUC values ranging from 0.681 to 0.757 (DR) and 0.721–0.684 (glaucoma), remains dependent clinician oversight due to model performance variability across external datasets. Models like FLAIR and RetiZero, despite notable success in rare disease recognition, also highlight the need for human-in-the-loop verification to ensure contextual appropriateness in diagnosis.

### 3.4 Risk of bias in studies

**Fig. 3:**
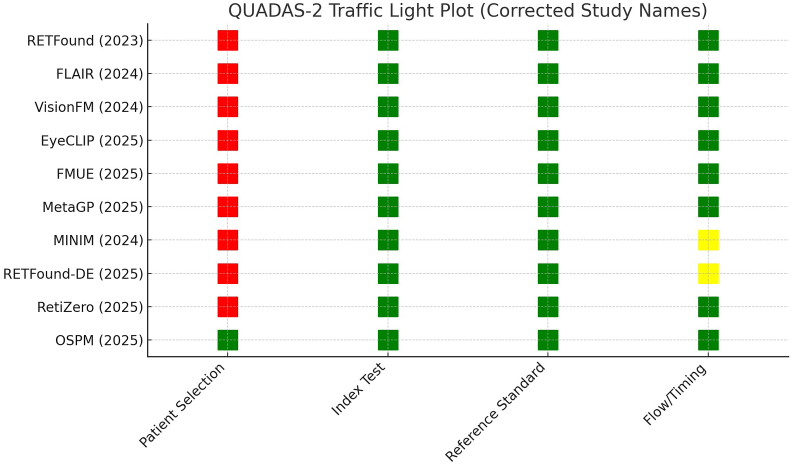
QUADAS-2 traffic light plot.

The QUADAS-2 assessment (Fig. 3) showed that the main source of potential bias across the ten included studies was in patient selection, with nine studies judged at high risk due to reliance on convenience sampling from public datasets or retrospective case series, while only the OSPM study used consecutive multi-center enrollment and was rated low risk. In contrast, the index test and reference standard domains were consistently low risk, as model outputs were generated automatically and compared against expert annotations or histopathology without review bias. For flow and timing, most studies were low risk, but MINIM and RETFound-DE were rated unclear because of potential inconsistencies when integrating synthetic and real data. Overall, these findings indicate that while the technical evaluation of foundation models in ophthalmology is generally robust, limitations in study populations, particularly non-consecutive and heterogeneous sampling, represent the predominant risk of bias and may constrain generalizability to real-world practice.

## 4 Discussion

The promising results of foundation models for eye diseases demonstrate their great potential to transform the landscape of diagnostic medicine. Synthesizing evidence from ten foundation model studies, the review reveals that both vision-based models and LLMs have demonstrated impressive diagnosis capabilities across a range of ophthalmological conditions, particularly retinal diseases, glaucoma, and ocular surface tumors. These models, pretrained on vast, often unlabelled datasets, are subsequently fine-tuned to tackle an array of downstream ophthalmic applications, including disease classification, progression tracking, and rare condition recognition. Most studies focused on high-prevalence pathologies such as diabetic retinopathy, age-related macular degeneration, and glaucoma, while others extended to rarer or underexplored conditions, demonstrating the breadth of use cases enabled by these systems. Collectively, these models offer significant opportunities to enhance diagnostic accuracy, streamline clinical efficiency, and expand access to expert-level interpretation in diverse clinical contexts.

### 4.1 Unifying multimodal data for enhanced diagnostic accuracy

A key advantage of foundation models lies in their capacity to process and integrate multimodal data sources, a feature that elevates their diagnostic breadth and depth.^23^ Several reviewed studies utilized vast amounts of ophthalmic data, including images from diverse modalities such as OCT, color fundus photography (CFP), and fluorescein fundus angiography (FFA), as well as clinical text and EHRs. For instance, EyeCLIP, a multimodal visual-language model, was trained on over 2.77 million ophthalmology images from 11 imaging modalities, augmented by partial clinical text, exemplifying the shift toward large-scale, multimodal learning architectures. This reflects significant progress in computational ophthalmology, enabling more comprehensive and adaptable diagnostic tools.

These foundation models are not confined to specific tasks or disease categories. In particular, models such as VisionFM and MetaGP demonstrated high generalization abilities, performing effectively across various diseases and clinical scenarios. VisionFM, a foundation model pretrained on 3.4 million ophthalmic images across multiple modalities, demonstrated strong generalization in diagnosing diseases like DR, glaucoma, and macular degeneration. It achieved high accuracy across diverse datasets, highlighting its adaptability to tasks ranging from screening to prognosis. This multimodal co-adaptation reflects a significant departure from prior siloed model designs and supports diagnostic personalization through data synthesis across anatomical, temporal, and contextual layers.

Beyond the ten core studies included, additional foundation models provide important insights into the evolution of ophthalmic FMs. For instance, EyeFM was trained on 10 million multimodal ophthalmic images and clinical reports, demonstrating robust performance in glaucoma detection.^24^ Its strength lies in integrating textual and imaging data, supporting cross-modal reasoning tasks. Similarly, multiple RETFound variants have been developed since the original 2023 RETFound model, including RETFound-DE (data-efficient pretraining with synthetic images), RETFound-Green (optimized for low-resource fundus screening with 75000 images), and RETFound-MEH (a 900000-image Moorfields dataset). These variants extend the generalizability of the RETFound framework, enabling adaptation to few-shot learning, domain shifts, and rare disease recognition. Together, EyeFM and the RETFound variants illustrate the rapid diversification of foundation model architectures in ophthalmology, highlighting a trend toward specialization while maintaining general-purpose adaptability.

### 4.2 Addressing rare and complex ophthalmic conditions

The ability of foundation models to handle rare and complex diseases is a particularly exciting development in ophthalmology. A few studies reviewed here focused on rare diseases or conditions with limited data availability. For example, MetaGP demonstrated robust diagnostic performance in rare disease and urgent care scenarios, such as Pompe disease and hereditary transthyretin amyloidosis, by integrating diverse datasets such as EHRs and multimodal imaging. RetiZero also showed remarkable zero-shot capabilities in identifying rare fundus diseases that are rarely represented in training data, such as Bietti crystalline dystrophy, chorioretinal coloboma, and punctate inner choroidopathy, achieving notably high top-3 accuracies and substantially outperforming prior models. EyeCLIP demonstrated improved diagnostic performance on 17 rare conditions such as birdshot retinochoroidopathy, central areolar choroidal dystrophy, choroidal melanoma, choroidal osteoma, cone dystrophy, Stargardt disease.

This advancement is particularly valuable in ophthalmology, where early and accurate diagnosis of less common or clinically complex conditions is essential to prevent irreversible vision loss. The ability of these models to identify subtle patterns and anomalies in medical images or clinical data makes them invaluable tools for clinicians, particularly in settings where access to expert care is limited.^25^

### 4.3 Demonstrating promising diagnostic accuracy and cross-domain robustness

Foundation models in ophthalmology have exhibited consistently high diagnostic performance across a wide spectrum of vision-threatening diseases. In the domain of retinal diseases, models achieved sensitivity ranging from 0.88 to 0.96 and specificity from 0.85 to 0.93. These performance metrics not only approach but in many instances exceed clinician-level diagnostic accuracy, particularly for DR and AMD-two of the leading causes of preventable blindness globally. Such performance highlights the clinical potential of these models in both screening and referral decision-making, where early identification and stratification are essential. Similarly, in the detection of glaucoma, a condition notoriously difficult to diagnose in its early stages due to the subtlety of anatomical changes, foundation models showed sensitivities ranging from 0.89 to 0.94 and specificities from 0.84 to 0.91. These figures surpass the typical performance metrics of traditional rule-based algorithms and some classical ML approaches, which often underperform in early-stage disease when functional deficits have not yet manifested. The ability of models such as RETFound, FMUE, and EyeCLIP to capture both structural and contextual features through multimodal input (e.g., OCT, fundus images, and clinical metadata) likely contributes to their superior predictive granularity.

An essential characteristic of foundation models, setting them apart from narrowly trained systems, is their demonstrated resilience when applied to external validation datasets. For example, FMUE, an OCT-based retinal model with embedded uncertainty estimation, retained an AUC of 0.989 on internal testing and performed comparably on external cohorts. Similarly, RETFound-DE, trained partially on synthetic data, achieved stable diagnostic performance across domains with only 2–16 labeled samples per class, illustrating the strength of its few-shot learning capabilities. These findings support the claim that foundation models not only learn visual and textual representations but also encode generalizable medical semantics that extend across imaging devices, healthcare institutions, and patient demographics.

### 4.4 Challenges in model optimization

However, it is critical to recognize that diagnostic accuracy in foundational models is not a static or absolute measure. Rather, it reflects a dynamic range that can evolve over time as the models are iteratively fine-tuned, retrained on new data distributions, or deployed in environments with different disease prevalence, annotation protocols, or clinical practices. Moreover, the operationalization of accuracy in real-world workflows is influenced by context-dependent factors, including user interface design, interpretability of results, integration into clinical decision support systems, and the training of end-users. Without alignment between algorithmic output and clinical utility, even high-accuracy models may fail to deliver meaningful benefit at the point of care.

What is particularly noteworthy of foundation models is the robustness of these models when validated on external datasets, highlighting their generalizability across different patient populations and settings.^26^ This is a crucial step toward ensuring the clinical applicability of these models in real-world environments and can continue to evolve as new data becomes available. Their evolving nature, while a strength in terms of adaptability, necessitates proactive model governance strategies to ensure sustained clinical relevance and safety in real-world settings.

### 4.5 Challenges in data quality, interpretability, and model generalization

Despite the impressive advancements, several challenges persist in the development and integration of foundation models into ophthalmology. Data quality and diversity are among the most significant concerns. Many studies relied on relatively homogeneous datasets, often sourced from single clinical centers or specific ethnic populations.^27^ This limitation could hinder the models’ ability to generalize to diverse patient demographics and real-world clinical scenarios.

Furthermore, algorithmic bias and overfitting remain persistent issues, particularly when models are trained on small or unbalanced datasets.^28^ This highlights the need for more diverse and representative data sources for training models to ensure that these models can perform reliably across various patient groups.^29^ While current diagnostic metrics are promising, they must be viewed as interim performance indicators. Continued validation across diverse and representative populations, particularly those historically underrepresented in ophthalmic AI research, will be essential to ensure fairness, reliability, and regulatory compliance.^30^^,^^31^ Additionally, longitudinal studies are warranted to assess the consistency of these models in real-time diagnostic pipelines, where variability in image quality, disease spectrum, and clinician feedback loops can impact model behavior. Establishing frameworks for post-deployment monitoring and adaptive recalibration will be key to sustaining diagnostic performance over time.

**Fig. 4:**
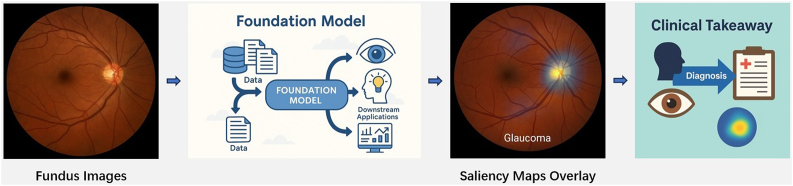
Worked example illustrating a fundus image with saliency overlay and corresponding clinical interpretation, demonstrating how interpretability tools can support decision-making in practice.

The interpretability of foundation models remains a significant and unresolved challenge, particularly in the context of clinical adoption. We further included a worked example (Fig. 4) demonstrating how saliency overlays on fundus images can be combined with clinical interpretation, illustrating the practical role of interpretability tools in guiding ophthalmic decision-making. While vision-based models have demonstrated high diagnostic capabilities, their inherent “black-box” nature often makes it difficult for clinicians to discern the rationale behind individual predictions.^32^ This opacity poses a substantial barrier to *epistemic trust*, a critical factor for the safe and effective deployment of AI in medicine. Clinicians must not only receive accurate outputs, but also be able to understand, scrutinize, and justify those outputs within the broader context of clinical decision-making.

To address this issue, a growing suite of explainable AI (XAI) tools has been proposed. Techniques such as SHAP (Shapley Additive Explanations) offer model-agnostic solutions for highlighting input features most influential to a model's output, thereby generating locally faithful surrogate explanations.^33^ These tools are particularly useful for high-dimensional imaging inputs, allowing ophthalmologists to visualize which anatomical structures, such as the macula, optic disc, or nerve fiber layer, most influenced a diagnosis. In addition to feature attribution methods, attention mechanisms embedded within model architectures can help surface implicit weighting strategies during training, particularly in vision-language models such as EyeCLIP or RetiZero. Such visual saliency maps and token-level relevance outputs provide clinicians with interpretable cues about both image and text contributions to the model's decision.^34^ More advanced forms of explanation, such as counterfactual reasoning, are increasingly being explored in the context of foundation models.^35^ Counterfactual explanations allow clinicians to ask "*what-if"* questions about model behavior, such as how a diagnosis might change if a specific retinal lesion were absent or if visual field metrics were altered. These counterfactuals not only aid transparency but also support error analysis, differential diagnosis, and model debugging. These advancements would increase clinician trust and facilitate the broader adoption of AI models in clinical workflows.

However, while these interpretability methods represent important steps forward, they are often developed independently of clinical context and remain underutilized in ophthalmology-specific applications. For these tools to support real-world diagnostic workflows, they must be embedded within clinician-facing interfaces, validated for clinical relevance, and co-designed with healthcare professionals. Future research should thus focus not only on improving algorithmic transparency but also on evaluating the usability, fidelity, and cognitive load of interpretability techniques within clinical decision support systems.

### 4.6 Clinical integration challenges: from architecture to application

The integration of foundation models into routine clinical practice remains a work in progress. Several studies noted the barriers to integration, including the need for substantial computational resources, compatibility with EHR systems, and clinician involvement in validating model outputs.^36^ Firstly, the computational burden is substantial. VisionFM and MINIM, for instance, require high-throughput computing to manage multimodal data fusion during inference, limiting their feasibility in real-time applications without edge-computing support. Secondly, EHR integration is fragmented. While MetaGP achieved a diagnostic score of 1.57 (vs. GPT-4's 0.93) by combining clinical records with imaging, the heterogeneity of EHR systems impedes seamless translation into hospital settings. Additionally, clinician validation is still essential. Models such as EyeCLIP, despite few-shot learning capacities, yielded AUC ranges of 0.681–0.757 for diabetic retinopathy, reinforcing the need for human-in-the-loop mechanisms for safe clinical implementation. To contextualize these gaps, Table 3 outlines the core architectural and deployment differences between traditional ophthalmic models and foundation models. As the table illustrates, foundation models offer a scalable and unified architecture, better suited for environments with evolving diagnostic needs and heterogeneous data. However, they simultaneously introduce novel demands in terms of governance, explainability, and clinical co-adaptation.

**Table 3:** Conceptual comparison of traditional models and foundation models in ophthalmology.

Aspect	Traditional Models	Foundation Models
Training Data Source	Task-specific labeled datasets	Large-scale unlabelled or weakly labeled data
Label Dependency	High (manual expert annotation required)	Low (supports few-/zero-shot learning)
Model Architecture	Independent models per task	Unified backbone with modular fine-tuning
Training Strategy	Train from scratch for each application	Pretrain once, fine-tune for downstream tasks
Task Adaptation	Poor (requires retraining)	Strong (supports cross-domain generalization)
Scalability	Low (siloed per-task deployment)	High (one model across multiple clinical tasks)
Computational Efficiency	Low (duplicate model development and training)	Higher (shared backbone; economies of scale)
Generalizability	Often limited to training domain	High; validated on external datasets
Clinical Integration Potential	Limited due to fragmented deployment pipelines	Promising, contingent on transparency, interpretability, and oversight

Note: This framework is derived from a synthesis of current literature on medical AI systems and reflects the expert consensus of the authors based on the studies reviewed in this systematic analysis.

To move foundation models toward routine use, several practical steps are required: (1) develop minimum integration layers to fit existing workflows; (2) optimize models for limited GPU and battery resources to enable use with portable fundus cameras; (3) ensure interoperability with PACS/EHR systems; (4) set fail-safe thresholds so that uncertain cases are referred to clinicians; and (5) implement continuous post-deployment monitoring for drift and fairness.

### 4.7 Ethical and regulatory frontiers

Foundational progress must also contend with ethical and regulatory realities. Regulatory approval, especially in high-stakes clinical applications such as surgery outcome prediction and diagnostic decision-making, was highlighted as a critical factor for successful deployment.^37^ The opaque decision-making processes inherent to many deep models risk eroding clinician trust, particularly in ophthalmology, where diagnostic interpretation is traditionally image-centric and consultative. Incorporating explainability methods such as SHAP, attention mechanisms, or counterfactual visualization can help bridge this gap. Moreover, future regulatory alignment with initiatives like the European Union (EU)'s AI Act, the FDA's Good Machine Learning Practices (GMLP), and ISO/IEC 23894 for AI risk management will be pivotal to ensure safety, fairness, and accountability in clinical use.^30^

While many foundation models have demonstrated promising results in research settings, few have been fully deployed in clinical environments. This gap between research and clinical application reflects the challenges of incorporating AI into daily practice, where real-time decision-making and collaboration with healthcare professionals are essential.^38^

### 4.8 Fairness, accessibility, and equity

An important limitation of current foundation models lies in fairness and accessibility. While overall AUCs and accuracies appear high, several recent studies demonstrate that such headline metrics can conceal significant disparities across devices, demographic groups, and minority populations. For example, portable fundus cameras and diverse acquisition settings, can expand access but also introduce domain shifts that foundation models may fail to generalize to Ref. 39. Similarly, demographic fairness remains a challenge: a study showed measurable performance gaps by sex and age, even in high-performing models.^40^ Moreover, the Harvard "Glaucoma Fairness" dataset documented substantial sensitivity differences across racial/ethnic groups, and proposed Fair Identity Normalization as a mitigation strategy.^41^ These findings underscore that models pretrained primarily on high-resource datasets may perform poorly in underserved settings with different imaging devices, demographic distributions, or disease prevalence. Without deliberate validation and adaptation, deploying FMs risks exacerbating inequities in ophthalmic care rather than reducing them. Therefore, future research must prioritize targeted external validation, inclusion of underrepresented populations, and fairness-aware training strategies to ensure safe and equitable deployment of foundation models in ophthalmology.

### 4.9 Future directions

The findings of this review point to the remarkable promise of foundation models in transforming ophthalmic diagnostics and clinical decision-making, particularly for improving diagnostic accuracy, accelerating disease detection, and enabling the identification of rare and complex conditions. However, to translate this promise into real-world impact, several critical challenges must be addressed. Chief among these are issues related to data heterogeneity, algorithmic bias, model interpretability, and the practical integration of AI systems into clinical workflows. Without deliberate, interdisciplinary efforts to resolve these barriers, the clinical utility and ethical sustainability of foundation models may remain limited.

However, a limitation of this review is the possibility of missing studies due to our search relying on "foundation model" labels. Some relevant works may not have explicitly used this term or could have been published in less formal sources, such as conference proceedings (e.g., MICCAI, ISBI). Future updates could benefit from broader search terms and manual curation from these venues to capture additional studies.

Future research should focus on.

(1) Expanding and diversifying of training datasets to encompass broader demographic, geographic, and phenotypic variation. This will be essential not only for improving model generalizability but also for mitigating the risk of performance disparities across underrepresented populations.
(2) Developing intrinsically interpretable or post hoc explainable models is vital for fostering clinician trust and ensuring regulatory readiness. Such efforts should include the co-design of user-centric explanation tools that align with real-world clinical reasoning.
(3) Exploring novel ways to enhance data efficiency, reducing the reliance on vast annotated datasets. These data-efficient training strategies, including few-shot learning, synthetic data augmentation, and self-supervised learning frameworks, should be systematically explored and rigorously validated within ophthalmology-specific contexts to ensure their effectiveness in reducing annotation burdens, enhancing model adaptability, and enabling scalable deployment across diverse clinical settings.
(4) Ensuring that models meet regulatory standards for clinical use and are capable of integration into real-world clinical environments. Rigorous attention must be paid to regulatory alignment, ensuring that foundation models meet evolving standards for safety, transparency, and post-deployment monitoring, as outlined in global frameworks such as the EU AI Act and FDA's GMLP guidelines.

These efforts will not only enhance the efficacy of foundation models in ophthalmology but will also ensure their safe and effective integration into clinical practice, ultimately improving patient outcomes and transforming the field of ophthalmic care.

## 5 Conclusions

This systematic review highlights the significant potential of foundation models in ophthalmology, particularly for improving the diagnosis of retinal diseases, glaucoma, and ocular surface tumors. These models, leveraging multimodal data, demonstrate high diagnostic accuracy and robustness across diverse ophthalmic conditions. However, challenges such as data quality, model interpretability, and integration into clinical workflows remain. To fully realize the potential of foundation models in ophthalmology, further research is needed to enhance model generalization, transparency, and clinical applicability. With continued advancements, foundation models would transform diagnostic practices and improve patient outcomes in ophthalmology.

## Study ​approval

Not Applicable.

## Author contributions

KJ led the conceptualization, formal analysis, investigation, and drafting of the original manuscript. TY contributed to methodology, validation, and data interpretation. GY, ZG, KZL, YZ, DS, and MW were primarily involved in reviewing and revising the manuscript, providing critical feedback to refine its content. PG contributed to reviewing the manuscript and improving methodological aspects. AG contributed to supervision, manuscript review, and critical editing.

## Funding

This work has been financially supported by 10.13039/501100001809Natural Science Foundation of China (grant number 82201195).

## Declaration of competing interest

The authors declare that they have no known competing financial interests or personal relationships that could have appeared to influence the work reported in this paper.

## Abbreviations

AI: artificial intelligence
DR: diabetic retinopathy
AMD: age-related macular degeneration
OCT: optical coherence tomography
CFP: color fundus photography
CNN: convolutional neural network
EHR: electronic health record
AUC: area under the curve
OST: ocular surface tumors
FLAIR: Foundation Language-Image Model of the Retina
MetaGP: Generative Foundation Model Integrating Electronic Health Records and Multimodal Imaging
RETFound: Retinal Foundation Model
VisionFM: Vision Foundation Model
RetiZero: Knowledge-Rich Vision-Language Model for Fundus Diseases
