# Supplementary material for: A systematic review of vision and vision-language foundation models in ophthalmology
Source: Adv Ophthalmol Pract Res. 2025 Oct 24;6(1):8–19. doi: 10.1016/j.aopr.2025.10.004 (PMC12794246; doi:10.1016/j.aopr.2025.10.004)
Supplement: Multimedia component 2 [file mmc2.docx]

Supplementary

Table S1. Exclusion Reasons.

| **Stage of Exclusion** | **Reason for Exclusion** | **Number of Articles Excluded** |
| --- | --- | --- |
| Before screening | Duplicate records | 394 |
| Before screening | Not written in English | 12 |
| Title/Abstract screening | Irrelevant to topic (not ophthalmology / not foundation model) | 8150 |
| Full-text retrieval | Report not retrievable (conference abstract only, inaccessible full text) | 300 |
| Full-text eligibility | Non-original research (reviews, opinions, editorials) | 43 |
| **Final included** | – | **10** |

Table S2. QUADAS-2 assessment.

| **Study** | **Patient Selection** | **Index Test** | **Reference Standard** | **Flow/Timing** |
| --- | --- | --- | --- | --- |
| **RETFound (2023)** | High | Low | Low | Low |
| **FLAIR (2024)** | High | Low | Low | Low |
| **VisionFM (2024)** | High | Low | Low | Low |
| **EyeCLIP (2025)** | High | Low | Low | Low |
| **FMUE (2025)** | High | Low | Low | Low |
| **MetaGP (2025)** | High | Low | Low | Low |
| **MINIM (2024)** | High | Low | Low | Unclear |
| **RETFound-DE (2025)** | High | Low | Low | Unclear |
| **RetiZero (2025)** | High | Low | Low | Low |
| **OSPM (2025)** | Low | Low | Low | Low |
